# Clinical *Aureobasidium* Isolates Are More Fungicide Sensitive than Many Agricultural Isolates

**DOI:** 10.1128/spectrum.05299-22

**Published:** 2023-03-21

**Authors:** Electine Magoye, Lukas Nägeli, Andreas Bühlmann, Maja Hilber-Bodmer, Peter Keller, Konrad Mühlethaler, Arnaud Riat, Jacques Schrenzel, Florian M. Freimoser

**Affiliations:** a Agroscope, Research Division Plant Protection, Nyon, Switzerland; b Agroscope, Research Division Food Microbial Systems, Wädenswil, Switzerland; c University of Bern, Institute for Infectious Diseases, Bern, Switzerland; d Bacteriology Laboratory, Division of Laboratory Medicine, Department of Diagnostics, Geneva University Hospitals, Geneva, Switzerland; University of Iowa Hospitals and Clinics

**Keywords:** *Aureobasidium*, fungicide resistance

## Abstract

Fungicide applications in agriculture and medicine can promote the evolution of resistant, pathogenic fungi, which is a growing problem for disease management in both settings. Nonpathogenic mycobiota are also exposed to fungicides, may become tolerant, and could turn into agricultural or medical problems, for example, due to climate change or in immunocompromised individuals. However, quantitative data about fungicide sensitivity of environmental fungi is mostly lacking. *Aureobasidium* species are widely distributed and frequently isolated yeast-like fungi. One species, *A. pullulans*, is used as a biocontrol agent, but is also encountered in clinical samples, regularly. Here, we compared 16 clinical and 30 agricultural *Aureobasidium* isolates based on whole-genome data and by sensitivity testing with the 3 fungicides captan, cyprodinil, and difenoconazole. Our phylogenetic analyses determined that 7 of the 16 clinical isolates did not belong to the species *A. pullulans*. These isolates clustered with other *Aureobasidium* species, including *A. melanogenum*, a recently separated species that expresses virulence traits that are mostly lacking in *A. pullulans*. Interestingly, the clinical *Aureobasidium* isolates were significantly more fungicide sensitive than many isolates from agricultural samples, which implies selection for fungicide tolerance of non-target fungi in agricultural ecosystems.

**IMPORTANCE** Environmental microbiota are regularly found in clinical samples and can cause disease, in particular, in immunocompromised individuals. Organisms of the genus *Aureobasidium* belonging to this group are highly abundant, and some species are even described as pathogens. Many *A. pullulans* isolates from agricultural samples are tolerant to different fungicides, and it seems inevitable that such strains will eventually appear in the clinics. Selection for fungicide tolerance would be particularly worrisome for species *A. melanogenum*, which is also found in the environment and exhibits virulence traits. Based on our observation and the strains tested here, clinical *Aureobasidium* isolates are still fungicide sensitive. We, therefore, suggest monitoring fungicide sensitivity in species, such as *A. pullulans* and *A. melanogenum*, and to consider the development of fungicide tolerance in the evaluation process of fungicides.

## OBSERVATION

Antifungal compounds are widely applied in agricultural and clinical settings to control fungal diseases. However, fungicide applications in the field or clinics are not specifically targeted to the pathogens but hit all mycobiota equally. We could even argue that fungicide applications predominantly affect non-target organisms, since pathogenic species usually comprise only a small proportion of the total mycobiome. The extensive use of antifungals, thus, poses strong selection pressure on target and non-target populations of fungi, and can lead to the development of resistance in both groups (1–3). *Aureobasidium pullulans* is an extremotolerant, yeast-like fungus that is highly abundant in most habitats (4, 5). Since it is one of the most frequently isolated fungal species, it is also regularly found in clinical samples, even though it likely does not cause disease in humans (6, 7). As we have shown previously, some agricultural *A. pullulans* isolates are highly tolerant to the antifungal agents captan (CPN), cyprodinil (CYP), and difenoconazole (DFN), which are commonly used in agriculture (8). CPN belongs to the phthalimide group of fungicides, which have been reported to act on thiol groups, and pose a low risk for resistance development (9, 10). CYP is an anilinopyrimidine that inhibits the biosynthesis of sulfur-containing amino acids and their precursors (e.g., methionine, cysteine, cystathionine, and homocysteine) (11). DFN is an azole, which is a broad-spectrum fungicide that is widely used in agriculture and clinics (12). Compounds of this class target, the cytochrome P450 enzyme lanosterol 14-α-demethylase (coded for by the *ERG11* or *CYP51A* gene), and resistance to azoles is widespread and well documented (13, 14).

We have previously described highly fungicide tolerant *A. pullulans* isolates from agricultural samples (8), but it was not clear if this low sensitivity is a general characteristic of this species, or has been selected for by repeated fungicide exposure, which would represent a largely neglected and unintended side effect of fungicide use. To better understand fungicide sensitivity in *A. pullulans*, 16 clinical isolates were obtained from Swiss hospitals or the Westerdijk fungal culture collection (Table S1). Genome analyses and sensitivity assays were used to compare these strains with 30 isolates from agricultural soil and plant samples (from apple orchards that were not treated with fungicides) (8).

**FIG 1:**
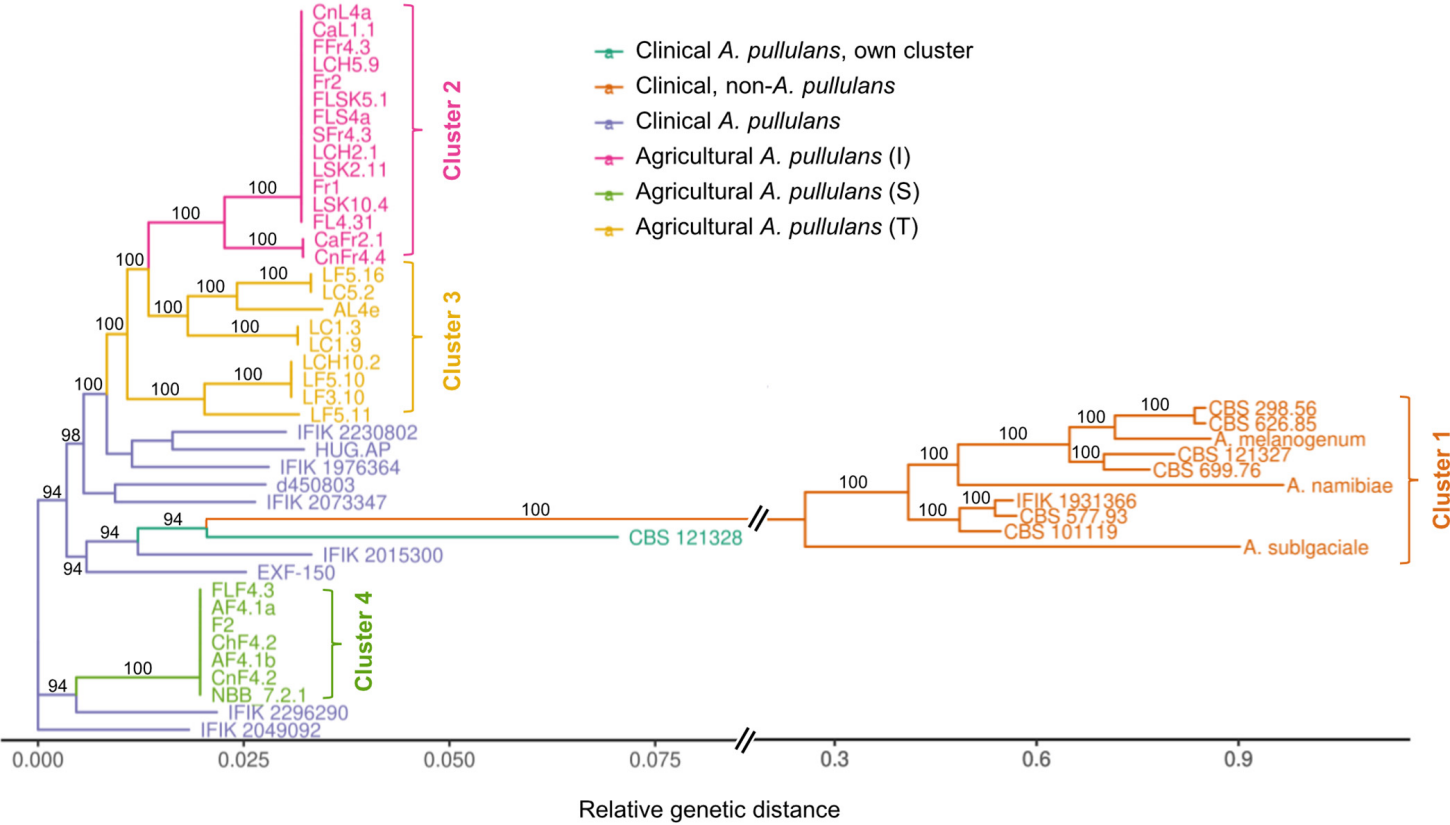
SNP-based phylogeny of 46 *Aureobasidium* strains from agricultural and clinical samples. Many clinical *Aureobasidium* isolates cluster more closely with other *Aureobasidium* species than with *A. pullulans*. As references, the analysis included 5 genomes of the 4 *Aureobasidium* species *A. pullulans* (NBB 7.2.1 and EXF-150), *A. namibiae* (EXF-3398), *A. melanogenum* (CBS 110374), and *A. subglaciale* (EXF-2481) (7, 15). The *x* axis represents the relative distances between the taxa. The *x* axis is discontinuous, as is indicated by the double line to fit the taxa of the genetically distant cluster 1. The numbers of the branches indicate bootstrap values of 100 bootstrap replicates and values <90 are not shown.

Based on our phylogenetic analysis, most isolates exhibited short relative distances to each other or were almost identical (Fig. 1). Only cluster 1, comprised of reference species and 7 of our strains (i.e., CBS 626.85, CBS 298.56, CBS 699.76, CBS 121327, CBS 101119, CBS 577.93, and IFIK 1931366), showed a large relative distance to all other isolates. Interestingly, these 7 isolates all originated from clinical samples and clustered together with the non-*A. pullulans* reference strains *A. melanogenum*, *A. namibiae*, and *A. subglaciale*. Due to the large genetic distance between these non-*A. pullulans* isolates (cluster 1) and all other isolates, a subsequent hierarchical clustering was performed with only the *A. pullulans* sequences. Based on this analysis, 3 clusters with exclusively agricultural isolates were defined. These 3 clusters (2, 3, and 4 in Fig. 1) corresponded to the previously reported groupings based on fungicide sensitivity (e.g., intermediate [I], tolerant [T], or sensitive [S] isolates, respectively) (8). In contrast, the clinical *A. pullulans* isolates showed larger genetic distances with lower bootstrap support (<90), and did not all form a single clade. The clinical isolate CBS 121328 differed the most from the other *A. pullulans* isolates and formed its own cluster, but was an order of magnitude less divergent than the non-*A*. *pullulans* cluster 1 isolates. Since none of the clinical isolates fell into one of the well-resolved clusters of agricultural isolates, a nonagricultural pool of *A. pullulans* was likely the source of these isolates that were encountered in the clinics.

**FIG 2:**
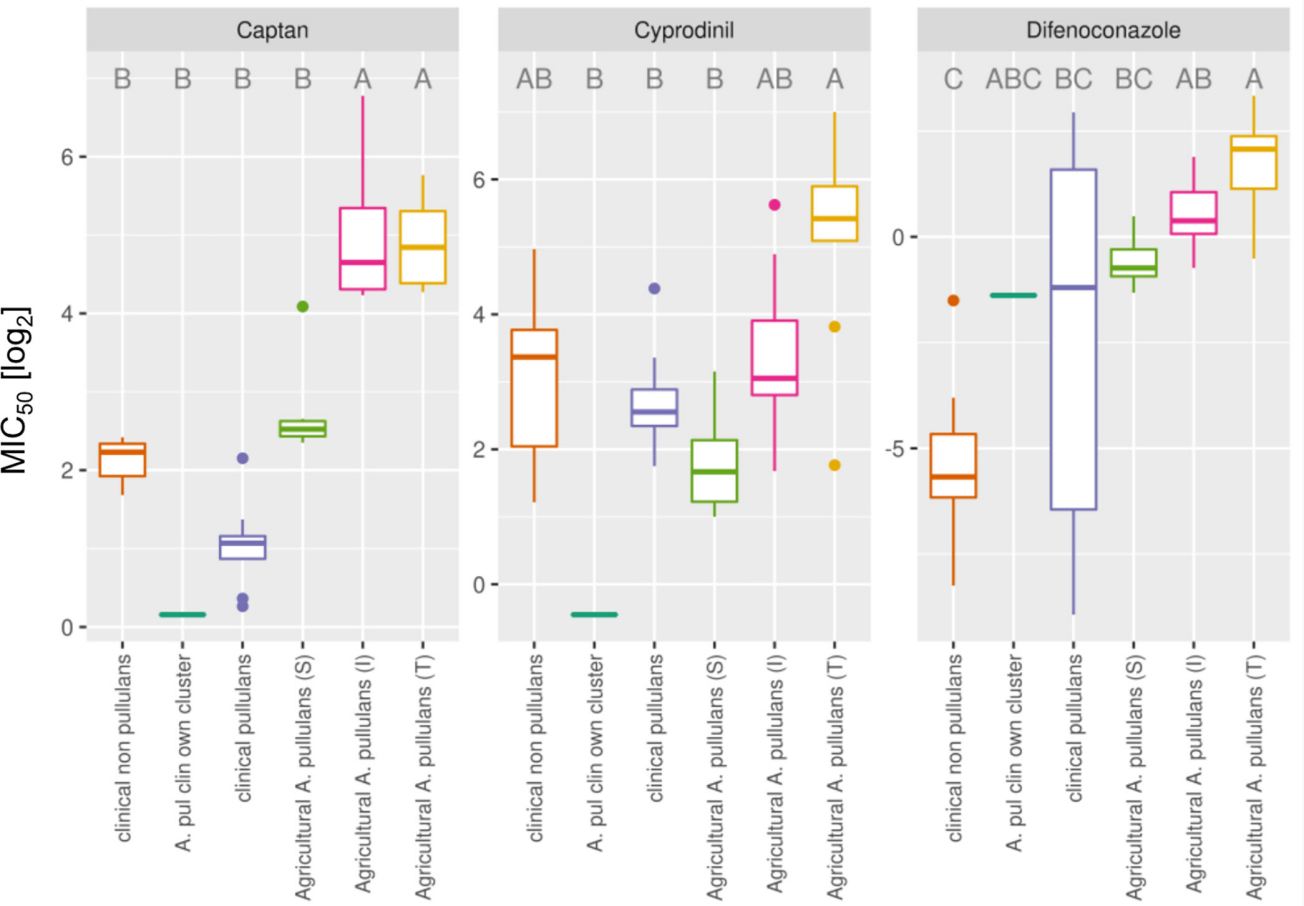
Clinical *Aureobasidium* isolates are more sensitive to captan, cyprodinil, and difenoconazole than many agricultural isolates. Boxplot of the MIC_50_ values (log_2_) for each group of isolates derived from the phylogenetic tree and for the 3 fungicides captan (CAN), cyprodinil (CYP), and difenoconazole (DFN). For 2 isolates that were not controlled at the maximum CYP concentration, 128 μg/mL (the highest concentration tested) was set as the MIC50 value. Different letters indicate statistical significance based on the Dunn’s-Test (P_adj_ ≤ 0.05).

To determine if fungicide sensitivity also correlated with genetic difference in this larger collection of isolates, and based on whole-genome data, the MIC_50_ values for CAN, CYP, and DFN were determined for all *Aureobasidium* isolates (Fig. 2). Overall, the cluster type (i.e., clinical *A. pullulans*, clinical non-*A. pullulans*, agricultural sensitive, agricultural intermediate, and agricultural tolerant) had a significant effect on the MIC_50_ values for all 3 fungicides (P_adj_ ≤ 0.05, Kruskal-Wallis-Test, see gitlab repository at the link below). Based on these experiments, the clinical isolates exhibited comparable MIC_50_ values as the sensitive, agricultural isolates for all 3 fungicides (blue and green box plots) (Fig. 2). The MIC_50_ values for these clinical and sensitive, agricultural *A. pullulans* isolates were significantly lower than those for the tolerant agricultural isolates for all 3 fungicides (P_adj_ ≤ 0.05, Dunn’s-Test). These results indicated that the tolerant phenotype of the agricultural *A. pullulans* was not a general property of the species, but likely selected for by the fungicide applications in agriculture.

In summary, all clinical *Aureobasidium* isolates, even those not belonging to the species *A. pullulans*, were sensitive to the fungicides tested here, and significantly more sensitive than many agricultural isolates. Consequently, the patients carrying these strains must have acquired them from populations of sensitive *Aureobasidium* stains and likely not from agricultural environments. The observation that many agricultural *A. pullulans* isolates were tolerant to the fungicides tested here, supports the conclusion that agricultural fungicide applications can select for tolerance in non-target fungi. Selection for fungicide tolerance would be particularly worrisome for the clinically relevant species *A. melanogenum*. This species is also found in the environment, but is known to exhibit virulence traits, such as growth at 37°C, siderophore production, or hemolytic activity that are mostly lacking in *A. pullulans* (6, 7). However, the *A. melanogenum* isolates tested here mostly exhibited high fungicide sensitivity. Overall, these results highlight that antifungal interventions affect the entire mycobiome, and can cause population shifts in non-target fungi due to the selection of tolerant genotypes.

## MATERIALS AND METHODS

### Strains.

All strains used or generated during this study are listed in Table S1. A total of 30 environmental *A. pullulans* strains have been characterized in the past (8). Further, 16 clinical *Aureobasidium* strains have been obtained from Swiss hospitals or the Westerdijk fungal culture collection (Table S1). All isolates were maintained on potato dextrose agar ([PDA]; Difco; Chemie Brunschwig AG) at 22°C.

### Genome sequencing.

Genomic DNA was extracted as previously described (15), and whole-genome sequencing (150 bp paired end reads) was performed at BGI Genomics. The reference isolate *A. pullulans* NBB 7.2.1 was originally isolated from a soil sample and characterized by genome, transcriptome, and secretome analyses (15, 16). For phylogenetic analysis, the genomes of *A. pullulans* EXF-150, *A. melanogenum* CBS 110374, *A. namibiae* EXF-3398, and *A. subglaciale* EXF-2481 were included (7) (all available at https://mycocosm.jgi.doe.gov). *De novo* genome assembly was performed with SPAdes by using the default options (17). A phylogenetic and molecular evolutionary (PHaME) analysis was carried out according to the developer’s instructions (18). The genetic distances calculated by PHaME were used to delineate 6 clusters, and to generate a phylogenetic tree using the RaxML method. The control files and R documentation for reproducing the analysis are all available at https://github.com/Luknaegeli/Apul_clinical, and sequencing reads have been deposited at NCBI in the bioproject PRJNA897728, under the accession number SAMN31580265 for the agricultural, and SAMN31634356 for the clinical isolates.

### Sensitivity testing.

The microbroth sensitivity assays of 16 clinical isolates (Table S1) to CYP, CPN, DFN, and their subsequent MIC_50_ causing reduction in growth to 50% values were determined as previously reported (8). Each growth experiment was repeated at least 3 times.
