# Supplementary material for: Clinical Aureobasidium Isolates Are More Fungicide Sensitive than Many Agricultural Isolates
Source: Microbiol Spectr. 2023 Mar 21;11(2):e05299-22. doi: 10.1128/spectrum.05299-22 (PMC10100788; doi:10.1128/spectrum.05299-22)
Supplement: Supplemental file 1 — Supplemental material. Download spectrum.05299-22-s0001.pdf, PDF file, 0.2 MB [file spectrum.05299-22-s0001.pdf]

Supplementary Table 1. All 46 *Aureobasidium* isolates that were used in this study. The MIC_{50} values for captan (CAN), cyprodinil (CYP), and difenoconazole (DFN) are indicated. The reference isoaltes used for sequence comparison in the molecular analyses are also listed. Strain d450803 was excluded as an outlier because it had overall reduced growth even in absence of the fungicides.

Nr.	Isolate	MIC_{50} [µg /ml] CAN	CYP	DFN	Sample	Reference
	HUG.AP	2.1	4.9	0.28	Cornea	This study
	IFIK 1931366	4.8	10.3	0.02	Thumb nail	This study
	IFIK 1976364	1.3	6.0	3.25	Dry skin from the foot	This study
	IFIK 2015300	4.5	5.2	0.01	Nail	This study
	IFIK 2049092	1.2	10.3	0.00	Dry skin from the foot	This study
	IFIK 2073347	2.6	3.4	0.68	Dry skin from the foot	This study
	IFIK 2230802	2.1	6.6	0.02	Dry skin from the foot	This study
	IFIK 2296290	2.1	20.9	2.93	Dry skin from the foot	This study
	d450803	2.1	5.8	7.69	Thumb	This study
	CBS 101119	3.3	5.0	0.02	Man BAL fluid, patient; Netherlands	CBS
	CBS 121327	5.4	2.3	0.35	Ear canal of child; Greece	CBS
	CBS 121328	1.1	0.7	0.38	Man onychomycosis; Greece	CBS
	CBS 298.56	4.4	3.4	0.00	Man, lymph node; USA	CBS
	CBS 577.93	5.3	31.3	0.02	Man bronchial-alveolar lavage, 3-year-old boy with chronic myeloid leukemia; Sweden	CBS
	CBS 626.85	4.7	14.1	0.01	Man peritoneal dialysis fluid; Australia	CBS
	CBS 699.76	3.2	13.2	0.07	Man thumb nail; Netherlands	CBS
211	F2	5.1	2.8	0.5	Apple flower	[8]
353	Fr1	22.5	9	1	Apple fruit	[8]
423	SFr4.3	25.1	7.9	2.4	Apple fruit	[8]
10	LSK 2.11	19.7	49.3	1.8	Apple leaf	[8]
100	FLSK 5.1	18.8	7.5	1.3	Apple leaf	[8]
9	LSK 10.4	41	18.9	2.4	Apple leaf	[8]
7	LCH 10.2	20.9	34.1	7.1	Apple leaf	[8]
8	LCH 5.9	51.5	11.9	1.3	Apple leaf	[8]
199	ChF4.2	6.3	2.2	0.9	Apple flower	[8]
6	LCH 2.1	44.7	29.7	3.7	Apple leaf	[8]
198	CaL1.1	19.1	20.6	1.1	Apple leaf	[8]
425	CaFr2.1	19.4	3.8	1.2	Apple fruit	[8]
5	LC 5.2	39.5	186	0.7	Apple leaf	[8]
	LC 1.9	38.4	59.6	5.2	Apple leaf	[8]
4	LC 1.3	50.8	50.5	4.2	Apple leaf	[8]
	LF 3.10	20.6	3.4	4.8	Apple leaf	[8]
18	LF 5.11	23.4	14.1	2.2	Apple leaf	[8]
393	FFr4.3	19.9	3.2	3.1	Apple fruit	[8]
19	LF 5.16	28.7	42.8	1.4	Apple leaf	[8]
30	LF 5.10	54.4	41.9	10.1	Apple leaf	[8]
	AF4.1b	17	8.9	0.4	Apple flower	[8]
	AL4e	19.3	1.45×10^{39}	2.2	Apple leaf	[8]
	AF4.1a	5.8	3.6	0.6	Apple flower	[8]
	CnF4.2	5.3	2	0.6	Apple flower	[8]
	CnL4a	21.4	6	1.7	Apple leaf	[8]
	CnFr4.4	33.1	9	0.9	Apple fruit	[8]
	FL4.31	40.2	8.3	1	Apple flower	[8]
	FLF 4.3	5.7	4.7	1.4	Apple flower	[8]
	FLS4a	109.6	6.6	1.8	Apple flower	[8]
